# Fenofibrate therapy in reducing the progression of diabetic retinopathy: revisiting the FIELD and ACCORD-EYE studies through the LENS trial

**DOI:** 10.1038/s41433-024-03410-9

**Published:** 2024-10-22

**Authors:** Maria S. Varughese, Ananth U. Nayak, Sarita Jacob

**Affiliations:** 1 University of Sheffield Medical School, Royal Hallamshire Hospital, Sheffield, UK; 2 University Hospitals of North Midlands NHS Trust, Stoke-on-Trent, North Staffordshire, UK; 3 School of Medicine, Faculty of Health, Keele University, North Staffordshire, UK; 4 University Hospitals Birmingham NHS Foundation Trust, Birmingham, UK; 5 Institute of Clinical Sciences, University of Birmingham, Birmingham, UK; 6 College of Life and Health Sciences, Aston University, Birmingham, UK; 7 Birmingham, Solihull and Black Country Diabetic Eye Screening Programme, Birmingham, UK

**Keywords:** Drug therapy, Education

The Fenofibrate Intervention and Event Lowering in Diabetes (FIELD) study was a multinational randomised trial of 9795 patients aged 50–75 years with type 2 diabetes mellitus (T2DM) [1]. The patients in the FIELD trial were randomly assigned to receive fenofibrate 200 mg/day (*n* = 4895) or matching placebo (*n* = 4900). During subsequent clinic visits, information regarding laser treatment for diabetic retinopathy (DR) which was deemed as a pre-determined tertiary endpoint of the main study was collated. Ophthalmologists who were masked to treatment allocation adjudicated on the defined instances of laser treatment for macular oedema, proliferative DR, or any other eye condition. In a sub-study of 1012 patients in the FIELD study, standardised retinal photography was done and photographs graded with Early Treatment Diabetic Retinopathy Study (ETDRS) criteria to decide on the cumulative incidence of DR and its component lesions.

It was observed that laser treatment was required more frequently in participants with sub-optimal glycaemic or blood pressure control than in those with better control of these factors. In patients with a higher burden of microvascular disease, the need for such treatment was not exaggerated by plasma lipid levels. The necessity for first laser treatment for all DR was noted to be significantly lesser in the fenofibrate group than in the placebo group (*n* = 164 [3.4%] patients on fenofibrate vs. *n* = 238 [4.9%] on placebo; with a hazard ratio [HR] 0.69, 95% CI 0.56–0.84; *p* = 0.0002; absolute risk reduction 1.5% [0.7–2.3]).

The results from the FIELD study demonstrated the significantly beneficial effects of fenofibrate in reducing the requirement for laser therapy, especially in preventing disease progression in patients with pre-existing DR [2]. Yet, there was a need for validating these findings within the setting of large prospective studies with progression of DR as the primary endpoint, which culminated in the ACCORD-EYE (Action to Control Cardiovascular Risk in Diabetes-EYE) study.

In the context of interpreting these two studies and their wider clinical implications, it must be remembered that DR was not the primary endpoint by design in either study. In fact it was a tertiary endpoint in FIELD, and DR endpoints were only recorded in a sub-study cohort of the ACCORD study population [3]. This is of particular relevance as DR continues to be the commonest microvascular complication of T2DM and is still a leading cause of blindness in the developed as well as developing world [4]. Previous trials have suggested that intensive glycaemic control and tight blood pressure strategies do protect against the development of DR, but the role of lipids in the pathophysiology of DR was much less pronounced.

FIELD and ACCORD-EYE together included 11,388 patients with T2DM, of whom 5701 were treated with fenofibrate (± statin) for up to 5 years [1, 3, 5]. Fenofibrate reduced first laser treatment by 31% (*p* = 0.0002), and the progression of DR with absolute reductions of 5.0% over 5 years (*p = 0.022, FIELD) and 3.7% over 4 years (*p* = 0.006, ACCORD-EYE) [5]. The benefit was greater in patients with DR than in those without pre-existing DR.

Optimising glycaemic control and intensive treatment of dyslipidaemia with fenofibrate and statin combination therapy had a synergistic effect and reduced the rate of progression of predominantly pre-existing DR, which was further substantiated with ongoing results of additional ocular outcomes from ACCORD-EYE [6, 7].

The presumed mechanism of action was thought to be due to the implications of fenofibrate treatment and its involvement in lipid and non-lipid pathways, including possibly the added beneficial effects on (a) apoptosis, (b) oxidative stress, (c) inflammation, (d) blood-retinal barrier breakdown, and (e) neuroprotection as well [3–5]. Experimental studies also demonstrated improved survival of retinal endothelial and pigment epithelial cells in conjunction with reduced stress signals under diabetic conditions and fenofibrate per se has improved retinal outcomes in rodent models of diabetes and in addition enhancing the impact on retinal neovascularization as well [8, 9]. Some smaller studies evaluating diabetic macular oedema not requiring immediate photocoagulation or intraocular treatment with adequate diabetes and blood pressure control were conducted. Patients received either fenofibrate or placebo once daily for 12 months with total macular volume (TMV) measured in the worse eye and all eligible eyes with time-domain optical coherence tomography at baseline and quarterly thereafter showed a modest improvement in TMV [10]. Notable reductions in central macular thickness (CMT) measurements have been reported over a duration of 6 months with fenofibrate treatment [11]. It is thought that downregulation of basement membrane components by fenofibrate may have a protective effect against outer blood-retinal barrier leakage associated with DR [12]. Another observation has been the effect of fenofibrate on increasing circulating haematopoietic stem/progenitor cells (HSPCs), which have implications for vascular endothelial properties that has been shown to have a protective effect on DR [13].

The question of whether fenofibrate should be routinely used as a reasonable standard treatment for diabetic microvascular disease then ensued. This became even more important, given the global public health burden of disease and burden of cost of disease with respect to treating sight threatening complications of DR [14, 15]. Although prior optimisation of glycaemic control continued to show reduced DR progression, when the ACCORD study ended [16], the benefit of fenofibrate did not persistently sustain on follow up studies.

During the combined evaluative assessment of large cardiovascular trials conducted, fenofibrate treatment decreased the need for laser treatment by over 20% compared with placebo [17]. In order to receive worldwide regulatory endorsement, to allow for widespread use of this generically available and simple treatment to reduce the risk of progressive DR and maculopathy, it was necessary to conduct randomized trials precisely designed to primarily test its impact on diabetic eye outcomes.

The Lowering Events in Non-proliferative Retinopathy in Scotland (LENS) trial (NCT03439345; 1150 participants with T1DM or T2DM) was designed as a streamlined randomised double-masked placebo-controlled trial, based in Scotland, assessing whether treatment with fenofibrate (145 mg tablet daily or, in the context of impaired renal function, on alternate days) in people with early DR reduces progression to referable DR (defined in National Health Service [NHS] Scotland’s Diabetic Eye Screening grading scheme as referable background or proliferative DR, or referable maculopathy in either eye) or treatment with retinal laser, intravitreal injections or vitrectomy [18].

In the fenofibrate group compared with the placebo group, the frequencies for any progression of DR or maculopathy were *n* = 185 (32.1%) vs. *n* = 231 (40.2%); hazard ratio, 0.74; 95% CI, 0.61 to 0.90 and for the development of macular oedema were *n* = 22 (3.8%) vs. *n* = 43 (7.5%); hazard ratio, 0.50; 95% CI, 0.30 to 0.84 [19]. These results are promising as fenofibrate led to a 27% lower risk of progression of, or treatment for, DR or maculopathy compared with placebo over 4 years [20].

Treatment with fenofibrate appeared equally effective in participants with T1DM and T2DM in the LENS trial [19, 20].

Current treatments primarily focus on advanced stages of DR, and there is most certainly a critical need for earlier cost-effective therapies that can intervene sooner than later in the pathophysiology of the progression of DR [20]. The results of the LENS trial are in keeping with more recent evidence emerging yet again from the existing and available data so far from the follow up of previous FIELD and ACCORD-EYE studies as well as large robust population based evaluation studies [21–25]. We will have to await further randomised control trials that involve more diverse populations as the LENS trial cohort were largely White (97.7%) [19]. More data is needed to ensure that the findings are applicable across different ethnic groups., greater visualised area of the retina, and more specific assessment of retinal morphology as the LENS trial, did not use optical coherence tomography, without which diabetic macular oedema could be missed [20].

The Fenofibrate and Microvascular Events in Type 1 Diabetes Eye (FAME 1 Eye) trial (NCT01320345; 450 participants with T1DM) from Australia [26], and the Randomized Clinical Trial Evaluating Fenofibrate for Prevention of Diabetic Retinopathy Worsening (NCT04661358; 910 participants with T1DM or T2DM Protocol AF) by the Diabetic Retinopathy Clinical Research group in the USA [27], will help to robustly establish and consolidate the longer-term impact of fenofibrate treatment on slowing the progression of pre-existing DR in people with both T1DM and T2DM [17–20].
